# Supplementary material for: Bayesian gamma-negative binomial modeling of single-cell RNA sequencing data
Source: BMC Genomics. 2020 Sep 9;21(Suppl 9):585. doi: 10.1186/s12864-020-06938-8 (PMC7487589; doi:10.1186/s12864-020-06938-8)
Supplement: Supplementary file 1 — Additional figures. [file 12864_2020_6938_MOESM1_ESM.pdf]

Supplementary: Bayesian Gamma-Negative Binomial Modeling of Single-Cell RNA Sequencing Data

Siamak Zamani Dadaneh, Paul de Figueiredo, Sing-Hoi Sze, Mingyuan Zhou and Xi

November 4, 2019

1 Additional Figures

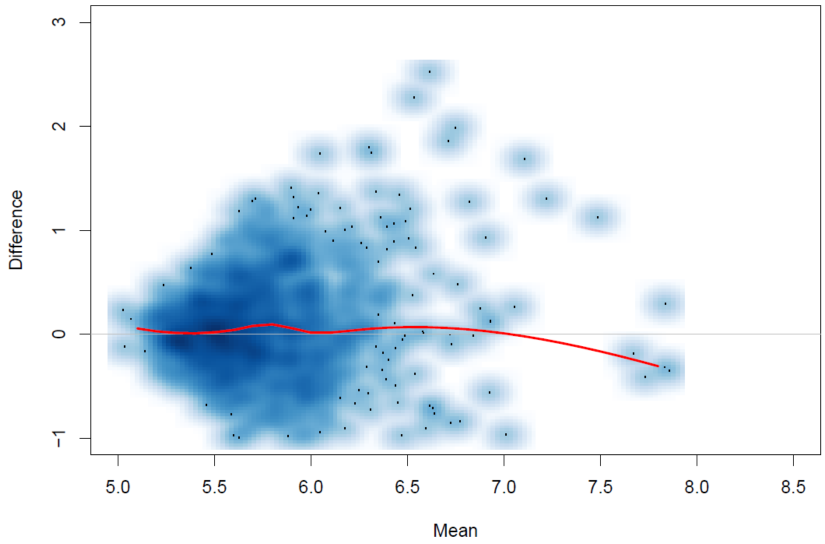

Figure 1: Mean-difference (MD) plot for V1 dataset. The solid red line represents the local regression fit to the data

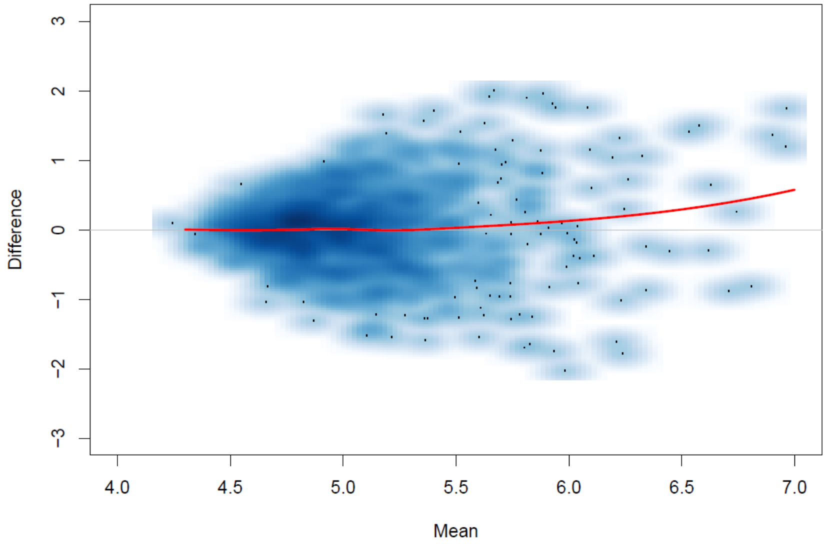

Figure 2: Mean-difference (MD) plot for mESC dataset. The solid red line represents the local regression fit to the data

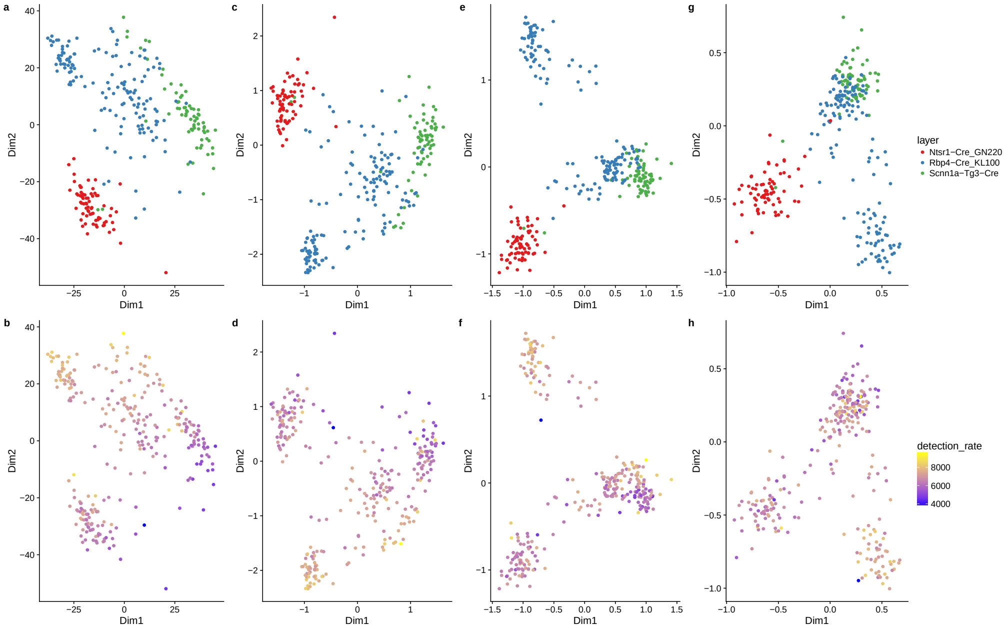

Figure 3: Low-dimensional representations of the V1 dataset. Panels correspond to (**a**) PCA (on total-count normalized data), (**b**) ZIFA (on total-count normalized data), (**c**) ZINB-WaVE, and (**d**) hGNB.

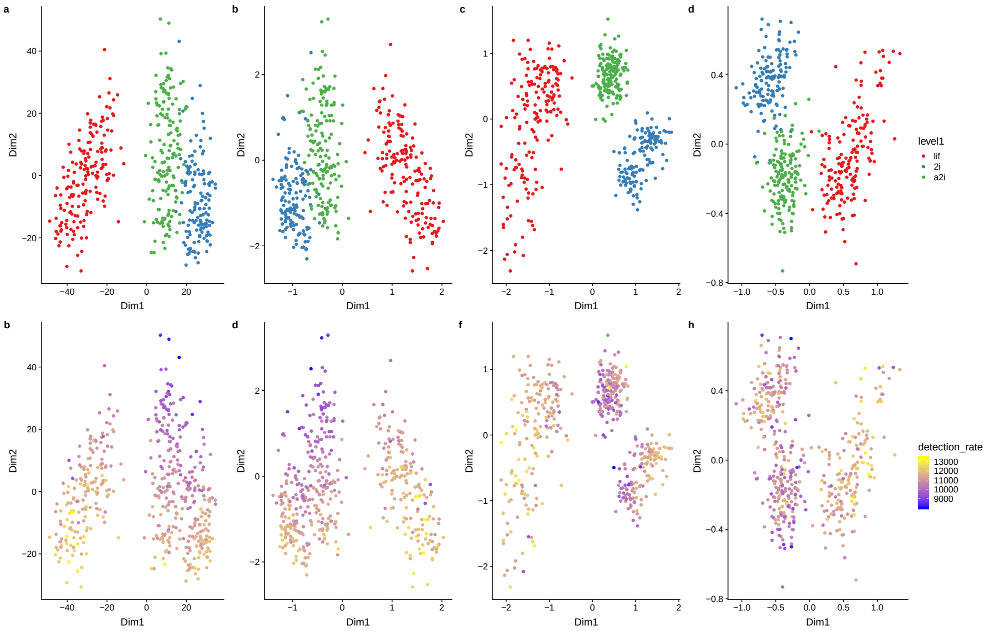

Figure 4: Low-dimensional representations of the mESC dataset. Panels correspond to (**a**) PCA (on total-count normalized data), (**b**) ZIFA (on total-count normalized data), (**c**) ZINB-WaVE, and (**d**) hGNB.
